# Variants in the 3' UTR of *General Transcription Factor IIF, polypeptide 2* affect female calving efficiency in Japanese Black cattle

**DOI:** 10.1186/1471-2156-14-41

**Published:** 2013-05-10

**Authors:** Shinji Sasaki, Takayuki Ibi, Toshio Watanabe, Tamako Matsuhashi, Shogo Ikeda, Yoshikazu Sugimoto

**Affiliations:** 1 Shirakawa Institute of Animal Genetics, Japan Livestock Technology Association, Odakura, Nishigo, Fukushima, 961-8061, Japan; 2 Graduate School of Environmental and Life Science, Okayama University, Tsushima-naka, Okayama, 700-8530, Japan; 3 Gifu Prefectural Livestock Research Institute, Kiyomi, Takayama, Gifu, 506-0101, Japan; 4 Cattle Breeding Development Institute of Kagoshima Prefecture, Osumi, So, Kagoshima, 899-8212, Japan; 5 Present address: National Livestock Breeding Center, Odakura, Nishigo, Fukushima, 961-8511, Japan

**Keywords:** Calving efficiency, Number of calves produced at 4 years of age (NCP_4_), Genome-wide association study, *General Transcription Factor IIF, polypeptide 2* (*GTF2F2*), Beef cattle

## Abstract

**Background:**

Calving efficiency can be described as the measure of a cow’s ability to produce viable offspring within a specific period of time. This trait is crucial in beef cattle because calves are necessary both for the production of beef and for heifer replacements. Recently, the number of calves produced at 4 years of age (NCP_4_) has been used to evaluate the calving efficiency of Japanese Black cattle. To identify variants associated with calving efficiency in Japanese Black cattle, we conducted a genome-wide association study (GWAS) using 688 animals with extreme NCP_4_ values selected from 15,225 animals.

**Results:**

We identified genetic variants on bovine chromosome 12 (BTA12) that were associated with NCP_4_. The *General Transcription Factor IIF, polypeptide 2* (*GTF2F2*), located in the 132 kbp-associated region, proved to be in strong linkage disequilibrium. We found 15 associated variants in the promoter and the 3' UTR regions. Consistent with this finding, transcripts of *GTF2F2* derived from the haplotype (*Q*) with the increased number of calves were 1.33-fold more abundant than *q*-derived transcripts. Furthermore, luciferase assays revealed that the activity of the 3' UTR, a region that includes nine SNPs, was higher in constructs with the *Q* haplotype than in those with the *q* haplotype by approximately 1.35-fold. In contrast, the activity of the promoter region did not differ between haplotypes. The association was replicated in an independent sample of 827 animals that were randomly selected from the remainder of the cohort from the same farms used in the GWAS. In the replicated population, the frequency of the *Q* haplotype is 0.313, and this haplotype accounts for 2.69% of the total phenotypic variance. The effect of the *Q* to *q* haplotype substitution on NCP_4_ was 0.054 calves. These findings suggest that variants in the 3' UTR of *GTF2F2* affect the level of *GTF2F2* mRNA, which is associated with calving efficiency.

**Conclusions:**

This GWAS has identified variants in the 3’ UTR of *GTF2F2* that were associated with the NCP_4_ of Japanese Black cattle, and this association was validated in an independent sample. The *Q* haplotype will be immediately useful in improving the calving efficiency of Japanese Black cattle.

## Background

**Figure 1:**
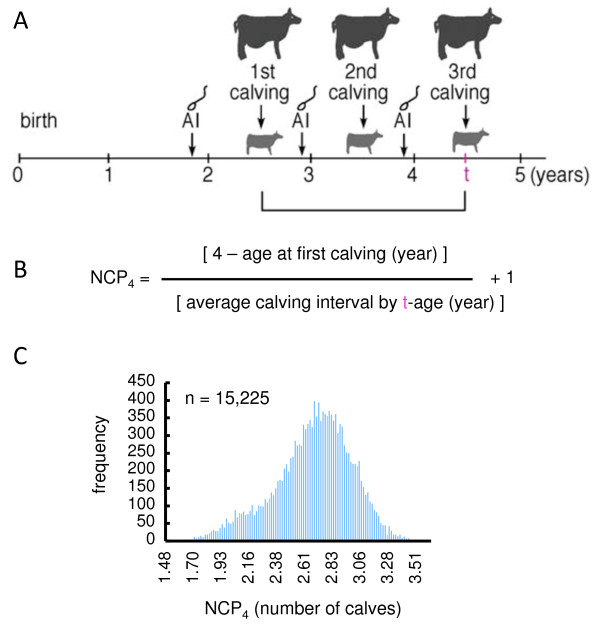
**Number of calves produced at 4 years of age (NCP**_**4**_**).** (**A**) Schematic representation of the reproductive cycle of Japanese Black cattle. At puberty, the heifer is artificially inseminated (AI) at the onset of estrus and becomes pregnant. After parturition (calving), the cow is again AI after the estrus cycle is re-established. Ideally, a cow produces a single calf each year. (**B**) The number of calves produced at 4 years of age (NCP_4_) is calculated from the following formula according to the method of the Wagyu Registration Association [2]; NCP_4_ = [4 - age at first calving (year)] / [average calving interval by t-age (year)] + 1. The t-age is the age at which the first calving occurs after the cow reaches 4 years of age. (**C**) The distribution of NCP_4_ in 15,225 cows in this study.

Calving efficiency is a major factor in determining the efficiency of beef cattle reproduction and directly connected to farm profitability because cow-calf producers sell their calves in calf markets in Japan. Recently, Oyama et al. developed a new index for calving efficiency, which is the measure of the number of calves produced at a specified age (NCP) [1]. Since 2008, the Wagyu Registry Association in Japan has been evaluating the calving efficiency of Japanese Black cattle nationwide according to the number of calves produced at 4 years of age (NCP_4_) [1], a slight modification of the original formula (Figure 1A,B) [2]. The heritability of this trait for Japanese Black cattle was estimated to be approximately 0.1 [1,2]. This level of heritability is relatively high compared to those of other reproductive traits, such as days open and calving interval, the heritability of which were estimated to be 0.047 in the population [3]. Furthermore, NCP_4_ maintains a high genetic correlation with subsequent calving performance [1], suggesting that NCP_4_ may be an effective index for genetic analysis and for predicting calving efficiency.

To identify and confirm variants associated with calving efficiency in Japanese Black cattle, we conducted a genome-wide association study (GWAS) with selective genotyping for NCP_4_ and a validation study of the association in an independent sample.

## Results

### A GWAS identified a quantitative trait locus (QTL) for NCP_4_ on bovine chromosome 12 (BTA12) in Japanese Black cattle

The heritability of NCP_4_ was estimated to be 0.11 using the numerator relationship matrix among 15,225 animals based on pedigree information, which was consistent with previous reports for Japanese Black cattle [1,2]. As shown in Figure 1C, the distribution was sufficiently wide to discriminate between higher- and lower-performance groups. We genotyped 357 cows from the upper extreme (93.3^rd^ percentile) of the distribution and 331 cows from the lower extreme (6.68^th^ percentile) among 15,225 records of NCP_4_ using BovineSNP50K BeadChips. The 33,303 SNPs on autosomes that fulfilled our criteria were used for the association study.

**Figure 2:**
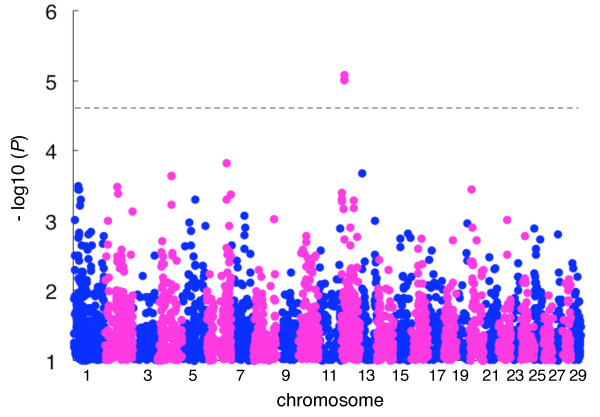
**Manhattan plot of the association of 33,303 SNPs with NCP**_**4 **_**in 688 Japanese Black cattle.** The chromosomes are distinguished with alternating colors (blue, odd numbers; red, even numbers). The chromosome number is indicated on the *x*-axis. The dashed line is the genome-wide significance threshold (−log10 (*P*) = 4.60) [5-7]. The vertical axis is broken for *P* values below -log10 (*1*).

The analysis was performed using EMMAX software [4], which is based on a linear mixed model approach using a genetic relationship matrix estimated by SNP genotypes to model the correlation between the phenotypes of the sample subjects. The genomic inflation factor (λGC) in this analysis was 0.9964, indicating that the sample was not stratified in the population and thus was appropriate for an association study. The quantile-quantile (Q-Q) plot showed that three SNPs deviated from the distribution under the null hypothesis (Additional file 1). In this study, we used a genome-wide significance threshold at the *P* < 2.5 × 10^-5^ level [5-7]. Three SNPs on BTA12 reached genome-wide significance (*P* < 8.4 × 10^-6^ - 9.7 × 10^-6^) (Figure 2, Table 1). The three SNPs were located within a 57-kbp window from 15,405,850 bp to 15,462,779 bp on BTA12 and proved to be in strong LD with each other (*D'*=1, *r*^*2*^ ranging from 0.95 to 0.99). The region consisted of three haplotypes defined by the genotypes of the three associated SNPs (Table 1) in the GWAS sample; the frequencies of the *Q* haplotype and *q* haplotype were 0.36 and 0.63, respectively.

**Table 1:** **SNPs with genome-wide significant associations with NCP**_**4 **_**on BTA12**

**BTA**	**SNP-id**	**Position (bp)_UMD3.1**	**Allele in upper extreme**	**Minor allele frequency in upper extreme**	**Minor allele frequency in lower extreme**	**Allele in lower extreme**	**Odds ratio**	***P*-value**
12	*ARS-BFGL-NGS-94479*	15405850	A	0.3599	0.2523	G	1.667	8.401E-06
12	*Hapmap39990-BTA-31570*	15441597	A	0.3697	0.2628	C	1.645	9.787E-06
12	*Hapmap36290-SCAFFOLD191599_16698*	15462779	A	0.3697	0.2628	G	1.645	9.787E-06

SNPs positions are based on the UMD3.1 assembly of the bovine genome.

The upper extremes and lower extremes correspond to NCP_4_ values above the 93.3^rd^ percentile and below the 6.68^th^ percentile, respectively.

**Figure 3:**
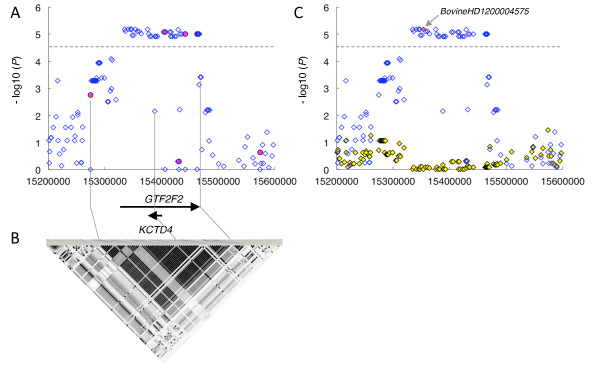
**Regional plot of the locus on BTA12 associated with NCP**_**4**_**.** (**A**) Regional plot of the locus on BTA12 associated with NCP_4_. SNPs from the 50K SNP chip are shown as red circles. The imputed SNPs are shown as unfilled blue diamonds. Genes and their directions of transcription are noted at the bottom of the plot. (**B**) The lower panels show estimates of the square of the correlation coefficient (*r*^*2*^) calculated for each pairwise comparison of SNPs. The *r*^*2*^ values are indicated by a black/white gradient: white represents *r*^*2*^ = 0, shades of gray represent 0 <*r*^*2*^ < 1, and black represents *r*^*2*^ = 1. (**C**) A conditioned analysis was performed by including a genotype of *BovineHD1200004575*, one of the most closely associated SNPs on BTA12, as a covariate in the model. A red filled diamond indicates the *BovineHD1200004575* (arrow)*.* The blue unfilled and yellow filled diamonds represent *P* values in –log10 scale before and after conditioning, respectively.

To define the region in more detail, the genotypes of 33,303 SNPs were imputed with haplotype information inferred from 586,812 SNPs in 1,041 Japanese Black cattle as a reference. The results showed that the average allelic matching error was 0.28%, and the average genotypic matching error was 0.55% across all of the chromosomes (Additional file 2), indicating that the imputation was highly accurate. Consequently, 35 SNP associations were detected within the 132-kbp window from 15,334,835 bp to 15,467,060 bp on BTA12 (*D'*=1, *r*^*2*^ ranging from 0.95 to 1.00) (Figure 3A,B, Additional file 3).

We then performed a conditioned analysis to ascertain whether there were any other associated SNPs of significance in the region. The genotype of one of the most closely associated SNPs, *BovineHD1200004575* (15,353,468 bp) on BTA12 (Figure 3C, Additional file 3), was individually included as a covariate in the linear mixed model. After conditioning, the associations of the other SNPs disappeared (Figure 3C, Additional file 3), indicating that the region contains a single QTL.

### Variants in the *GTF2F2* region were associated with NCP_4_

The LD region harbors two genes: *General Transcription Factor IIF*, *polypeptide 2* (*GTF2F2*) and *Potassium Channel Tetramerization domain containing 4* (*KCTD4*), which is located in intron 4 of *GTF2F2* and is transcribed in the opposite direction (Figure 3A). To detect association polymorphisms in *GTF2F2* and *KCTD4*, we sequenced all of the exons and upstream regions, beginning 3 kbp upstream of the start codon of each gene in three animals with *Q-* and *q-*homozygous haplotype that were defined by the genotypes of *BovineHD1200004575* (15,353,468 bp) and *BovineHD1200004611* (15,465,327 bp) (Additional files 3 and 4). In the *GTF2F2* region, we found one synonymous SNP in the coding region, five SNPs and one indel in the upstream region, and we found nine SNPs in the 3' UTR (Additional file 5). We also found a non-synonymous SNP (Ile 189 Val) and one indel in the region upstream of *KCTD4* (Additional file 5). To determine whether these variants of *GTF2F2* and *KCTD4* are associated with NCP_4_, we genotyped the variants in the GWAS samples and analyzed the association with NCP_4_ using EMMAX software with a genetic relationship matrix among animals. There was a total of 16 variants of the *GTF2F2* region that produced a highly significant signal (*P* = 4.1 × 10^-5^ - 5.28 × 10^-6^), whereas the two variants of *KCTD4* were not associated with NCP_4_ (*P* = 0.02 - 0.03). These association studies suggest that the causative variants are located within the *GTF2F2* region.

### Allelic imbalance level of *GTF2F2* mRNA

**Figure 4:**
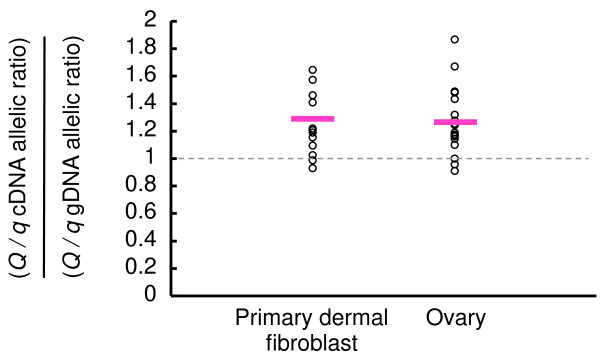
**The allelic imbalance test for levels of *GTF2F2 *mRNA in the heterozygotes.** The cDNA from primary dermal fibroblasts and ovaries and genomic (g) DNA from heterozygous animals was amplified using primers to *BovineHD1200004611* (15,465,327 bp) on BTA12, which is located in the exon of *GTF2F2* (Additional files 3 and 5). The PCR product was directly sequenced. Peak height at the SNP was quantified using PeakPicker 2 software [8]. The *y*-axis shows the ratio of the ratios of the peak height of the *Q* allele over the *q* allele in the cDNA and in the gDNA from the same animal. Red bars show the mean expression in primary dermal fibroblasts (n = 13, mean = 1.336) and ovaries (n =19, mean = 1.325). The *P* values for the difference between the ratios of the peak height of the *Q* allele over the q allele in the cDNA and the ratios of the peak height of the *Q* allele over the q allele in the gDNA were 0.002 (primary dermal fibroblast) and 0.0011 (ovary), respectively, as determined by *t*-test.

We observed that *GTF2F2* was expressed in the female reproductive organs as well as other tissues and cells, including fibroblasts (Additional file 6). Using an allelic imbalance test [8,9], we compared the relative abundance of *Q*- versus *q*-derived transcripts of *GTF2F2* in primary dermal fibroblasts (n = 13) and ovaries (n = 19) from heterozygotes. We isolated samples of genomic (g) DNA and cDNA derived from the same heterozygotes and amplified an SNP in the exon of *GTF2F2* (15,465,327 bp, Additional file 5), which is in strong LD with other *Q* alleles (*r*^*2*^ = 0.95 - 1). We then compared their allelic ratios using PeakPicker2 software [8]. The results revealed that the *Q*-derived transcripts of *GTF2F2* were 1.33-fold more abundant than *q*-derived transcripts in both primary dermal fibroblasts and ovaries (Figure 4).

### Variants in the 3' UTR of *GTF2F2* were involved in the allelic imbalance of the level of *GTF2F2* mRNA

**Figure 5:**
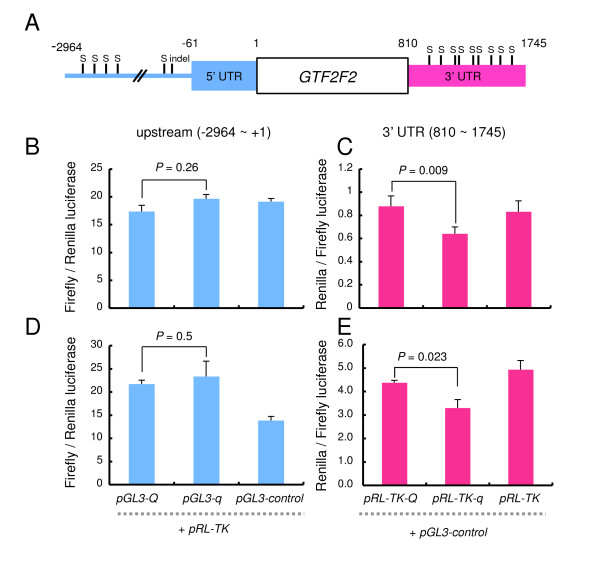
**Luciferase reporter assay for the 5' upstream region and the 3' UTR of *GTF2F2 *derived *Q *and *q *haplotypes.** (**A**) Schematic representation of the positions of variants in the 5' upstream region and the 3' UTR of *GTF2F2*. “S” and “indel” represent SNPs and indel, respectively. The detailed positions of the variants are shown in Additional file 5. The 5' upstream region (2,964 bp upstream from the start codon, blue) and the 3' UTR of *GTF2F2* (from 810 bp to 1,745 bp of mRNA, red) derived from the *Q* and *q* haplotypes were cloned into Firefly luciferase (*pGL3-Q* and *pGL3-q*) and Renilla luciferase (*pRL-TK-Q* and *pRL-TK-q*) vectors, respectively. The Firefly-to-Renilla luminescence ratios observed when cotransfecting HeLa cells (**B**) or bovine endometrial epithelial cells (**D**) with the indicated luciferase reporters were measured to evaluate the effects of the 5' upstream region (blue). The Renilla-to-Firefly luminescence ratios observed when cotransfecting HeLa cells (**C**) or bovine endometrial epithelial cells (**E**) with the indicated luciferase reporters were measured to evaluate the effects of the 3' UTR (red). Error bars represent the ± SEM obtained in triplicate from three experiments. *P* values determined by *t*-test are shown in the graph.

In the *GTF2F2* region, we detected five SNPs and one indel in the upstream region and nine SNPs in the 3' UTR (Figure 5A and Additional file 5). The variants in the promoter region and the 3' UTR may affect promoter activity or mRNA stability, and they may contribute to the allelic imbalance of level of *GTF2F2* mRNA. To determine whether the variants are involved in the level of *GTF2F2* mRNA, we cloned the promoter region, beginning 2,964 bp upstream of the start codon, and 935 bp of the 3' UTR from both the *Q* and *q* haplotypes into luciferase reporter constructs (Figure 5A and Additional file 5). We then transfected HeLa cells (Figure 5B,C) and primary bovine endometrial epithelial cells (Figure 5D,E), expressing endogenous *GTF2F2* (Additional file 6), with these constructs and measured the resulting luciferase activity 24 hr after transfection. The activity of the 3' UTR was approximately 1.35-fold higher for the *Q* constructs than for the *q* constructs; *t*-test, *P* = 0.009 and 0.023, respectively (Figure 5C,E). In contrast, similar levels of luciferase activity were stimulated by the promoter regions from both haplotypes (Figure 5B,D). These results suggest that the variants, including the nine SNPs in the 3' UTR of *GTF2F2*, affect the level of *GTF2F2* mRNA, which may lead to an allelic imbalance in the levels of *GTF2F2* mRNA.

### The associated SNP was replicated in an independent sample set

Cross-validation of the GWAS results from independent samples may decrease the rate of false positives. We examined whether the *Q* haplotype, defined by *BovineHD1200004575* (15,353,468 bp) and *BovineHD1200004611* (15,465,327 bp: the SNP is located in the 3’ UTR of *GTF2F2*) (Additional files 3, 4 and 5), is associated with NCP_4_ in an independent sample set. We genotyped 827 animals that were randomly selected from the remainder of the cohort from the same farms used in the GWAS (Additional file 7). The distribution of the phenotypic values of the samples was similar to that of the parent population (Figure 1C and Additional file 7). The result showed that the *Q*-homozygous haplotype (A-G) was significantly associated with NCP_4_ compared to the *q*-homozygous haplotype (G-A); Tukey-Kramer post-hoc test, *P* = 0.0207 (Table 2). The haplotype frequency of *Q* is 0.313, indicating that the haplotype is common in Japanese Black cattle. We fitted a linear mixed model to the NCP_4_ values in the additive model and used restricted maximum likelihood (REML) to estimate the variance explained by the haplotype. We estimated the proportion of phenotypic variance explained by the haplotype as 0.0269 (Table 2), indicating that the haplotype accounts for 27% of the total genetic variance. The *Q* to *q* haplotype substitution effect on NCP_4_ was 0.054 calves (Table 2).

**Table 2:** **The proportion of phenotypic variance attributed to haplotype associated with NCP**_**4**_

**BTA**	**Haplotype**	**Position (bp)_UMD3.1**	***Q *haplotype**	**Number of animals genotyped for the SNP**	***Q *haplotype frequency**	**Heritability (*h***^***2***^**)**	**Haplotype effect on total phenotypic variance**^**1**^	***Q *to *q *haplotype substitution effect (calves)**^**2**^	***P*-value**^**3**^
12	*BovineHD1200004575* and *BovineHD1200004611*	15353468 and 15465327	A-G	827	0.313	0.11	0.0269	0.054	0.0207

^1^The effect of the haplotype was estimated as the least square mean values of GLM analysis. The statistical model for GLM analysis included fixed effects for the farm, birth year and haplotype. The genetic variance explained by the haplotype was calculated based on estimates of the haplotype effect and the frequency of the haplotype [40]. Total genetic variance was estimated by MTDF-REML programs. The effect size of a haplotype was estimated as the proportion of genetic variance explained by the haplotype.

^2^The average NCP_4_ values for *QQ* and *qq* are 2.789 days and 2.683 calves.

^3^The results were tested by a one-way ANOVA, followed by a Tukey-Kramer test for multiple comparisons.

## Discussion

A substantial fraction of the environmental variance in NCP_4_ is attributed to farm variance [1], implying that minimizing the farm effect is particularly important in QTL mapping for NCP_4_. In this study, we collected a large number of reproductive records from 11 farms that were directly and uniformly managed by a single farming company.

In the current GWAS, we found that three SNPs in *GTF2F2* on BTA12 were associated with NCP_4_ in Japanese Black cattle (Figure 2 and Table 1). In Japanese Black cattle, the effective population size is small [10], and concomitantly, the number of haplotypes is limited. Accordingly, highly accurate imputation could be achieved with fine resolution using 1,041 animals with high-density genotypes as a reference (Additional file 2). The current imputation analysis revealed that additional 32 SNPs were also associated with NCP_4_ within a 132-kbp window, and they proved to be in strong LD with each other (Figure 3A,B and Additional file 3). Furthermore, a conditioned analysis revealed that a single QTL was present in the region (Figure 3C and Additional file 3).

We did not find any non-synonymous amino acid substitutions in the exons of *GTF2F2*. This observation is consistent with the increasing evidence that regulatory DNA variants controlling gene expression play a significant role in complex traits [9,11,12]. We compared the allelic mRNA ratio within the same heterozygous sample using an allelic imbalance test because it is more sensitive than quantitative RT-PCR and it is less affected by variation among samples [8,9]. We found larger amounts of *GTF2F2* mRNA from the *Q* allele (approximately 1.33-fold more) than *GTF2F2* mRNA bearing the *q* allele (Figure 4). In an attempt to identify the mechanism underlying this difference in the level of *GTF2F2* mRNA, we found that the level of luciferase activity driven by the 3' UTR of *GTF2F2* that included nine SNPs was higher for the *Q* haplotype than for the *q* haplotype (Figure 5C,E). These results suggest that the variants in the 3' UTR of *GTF2F2* may affect the level of *GTF2F2* mRNA, which, in turn, may influence calving efficiency. Recently accumulated evidence suggests that polymorphisms in the 3’ UTR can alter RNA secondary structure as well as the miRNA binding site [12-14]. At present, we have not found any regulatory RNA motifs or miRNA binding sites in the 3’ UTR of *GTF2F2* that differ between the *Q* and *q* haplotypes using databases such as RegRNA [15], Patrocle [16] or mirBase [17]. However, many SNPs associated with a broad range of complex traits alter the RNA structure [18]. Because the variants in the 3’ UTR influence its expression level, the variant should have the functional effect.

The level of luciferase activity driven by the 3' UTR of *GTF2F2* that included nine SNPs was approximately 1.35-fold higher for the *Q* haplotype than for the *q* haplotype (Figure 5C,E). This magnitude is comparable to that observed in the allelic imbalance test (Figure 4). Recently, causative variants affecting bovine stature have been identified in the promoter of *PLAG1*, and the level of *PLAG1* mRNA of the *Q* allele was slightly larger (approximately 1.36- to 1.81-fold) than that of the *q* allele in bone and muscle [9]. The quantitative difference between the *Q* and *q* animals was similar to that observed in our study, implying that a slight difference in the level of mRNA may have a large effect on phenotypic variance in some circumstances.

*GTF2F2* is ubiquitously expressed in bovine tissues (Additional file 6). GTF2F2*,* together with general transcription factor IIF, polypeptide 1 (GTF2F1), forms a heteromeric general transcription initiation factor (TFIIF) [19,20] that binds to DNA-dependent RNA polymerase II [21], the enzyme responsible for synthesizing mRNA. GTF2F2 has been shown to be necessary for initiation and elongation in gene transcription [22,23]. Although an increase in the expression of *GTF2F2* by allelic imbalance may lead to a slight augmentation of the activity of the polymerase, many genes driven by RNA polymerase II throughout the body could be widely affected and in turn could affect calving efficiency. Further studies about GTF2F2 could help elucidate the mechanisms underlying GTF2F2 function in calving efficiency.

Calving efficiency is influenced by several factors, including the onset of puberty; the ability to conceive, gestate, deliver a calf, re-enter the estrous cycle for the next pregnancy; and the duration of each of these reproductive phases. To date, particular attention has been focused on finding QTLs associated with each component, and several QTLs have been identified previously [6,7,24-29]. Although each trait is of interest in improving reproductive performance, comprehensive traits for calving efficiency, such as NCP_4_, may be effective indices for beef cattle breeding because NCP_4_ is directly calculated by measuring the ability of a cow to produce viable offspring.

Finally, we confirmed that the association was replicated in an independent, randomly selected sample (Table 2 and Additional file 7), which further strengthens the evidence for this location being the QTL. In the population, the frequency of the *Q* haplotype is 0.313 (Table 2), indicating that the haplotype is common in the Japanese Black cattle population. This QTL can be considered the major determinant for NCP_4_ in Japanese Black cattle, as this haplotype accounts for one fourth of the total genetic variance. Importantly, the *Q* haplotype does not have an adverse effect on calf production. Taken together, these markers will be useful for marker-assisted selection for NCP_4_ in Japanese Black cattle.

## Conclusions

This genome-wide association study demonstrated that the variants in the 3’ UTR of *GTF2F2* were associated with NCP_4_, and this association was validated in an independent sample. The use of the *Q* haplotype will be immediately beneficial in improving calving efficiency in Japanese Black cattle.

## Methods

### Ethics statement

All animal experiments were performed according to the Guidelines for the Care and Use of Laboratory Animals of Shirakawa Institute of Animal Genetics, and this research was approved by Shirakawa Institute of Animal Genetics Committee on Animal Research (H21-2).

### Collection of phenotypic data

Data were collected from farms managed by a large cooperative farming company raising Japanese Black cattle in Japan. The management system for Japanese Black cattle was described in a previous study [30,31]. The original data included 63,775 records of reproductive females born from 1992 to 2006. The data were selected using the following nine criteria for analysis: 1) data should be present for the cow from the first calving to the first calving after the age of 4 years; 2) the cow should not have had twins in parturition; 3) the cow should not have received any embryo transfers; 4) the cow should not have had abortions; 5) the length of all gestations should range from 261 to 310 days; 6) the calving interval should range from 276 to 730 days; 7) the age of the cow at the first calving should be less than 1,128 days; 8) the cow should be reared at a single farm; and 9) each breeding farm should have more than 10 records from each birth year.

After applying these selection criteria, the final dataset contained 15,225 records. The number of calves produced at 4 years of age (NCP_4_) was calculated from the following formula according to the Wagyu Registration Association [2]: NCP_4_ = [4 - age at first calving (year)] / [average calving interval by (t-age)] + 1 (Figure 1A,B). The t-age is the age at first calving after the age of 4 years. Each NCP_4_ value was corrected for the effects of farm and birth year. These effects were calculated as solutions of the REML procedure using MTDF-REML programs [32]. In this analysis, pedigree information was traced back two generations. The statistical model included fixed effects for the farm (174 farms) and birth year (1992 to June 2006). Direct genetic and residual effects were included as random effects.

### Selection of samples for GWAS and collection of DNA samples

Samples were selected from the 6.68% most extreme upper and lower performance values among 15,225 records of NCP_4_ from 11 farms directly managed by the cooperative farming company. To reduce population stratification, we selected fewer than five cows derived from a single sire in each extreme, resulting in 357 cows for the upper extreme and 331 cows for the lower extreme. The upper extreme included the offspring of 150 sires, and the lower extreme included the offspring of 128 sires. Whole blood was collected from each cow, and genomic DNA was isolated using the Easy-DNA kit (Invitrogen, cat# K1800-01).

### Genotypes and quality control

The DNA samples for the GWAS from 688 Japanese Black cattle were genotyped using the BovineSNP50K BeadChip (version 1, Illumina). Additionally, DNA samples for imputation from 1,041 Japanese Black cattle were genotyped using the BovineHD BeadChip (Illumina). The UMD3.1 assembly [33] was used to map the position of the SNPs. The SNP flanking sequences were provided by Illumina, lnc. (Additional file 8). The data were analyzed using PLINK v1.07 software [34]. The SNPs fulfilled our quality control criteria, which required a call rate greater than 99%, a minor allele frequency (MAF) greater than 0.01, and a chi-square test for Hardy-Weinberg equilibrium (HWE) *P* value greater than 0.001. Among the 777,962 SNPs on the BovineHD BeadChip, 586,812 autosomal SNPs fulfilled our quality control criteria. Among 54,001 SNPs on the BovineSNP50 BeadChip, 33,303 autosomal SNPs fulfilled our quality control and inclusion criteria, which required that the SNPs be included in the 586,812 SNPs on the BovineHD BeadChip.

### GWAS for NCP_4_

An association analysis was performed for the 688 samples using EMMAX software [4] based on a linear mixed model with a genetic relationship matrix. The software carried out the tests in the spirit of Armitage using a simple standard linear regression framework with 0–1 quantitative response variables representing the upper and lower extremes. In a conditioned analysis, the genotype of the SNP associated with NCP_4_ was included as a covariate.

We used the Lander and Kruglyak method to identify genome-wide significance thresholds [5]. This method accounts for the effective number of tests within the genome. The genome-wide significance level is μ(*T*) = [*C* + 2ρ*GT*^2^]α(*T*), where *T* is the value of the test statistic, *C* is the number of chromosomes, *G* is the length of the genome in Morgans (excluding the sex chromosomes), and ρ is the expected rate of recombination, which was taken as 2 [6,7]. The point-wise significance level of exceeding *T* is α(*T*). The genome-wide 5% significance threshold corresponds to 2.5 × 10^-5^.

### Linkage disequilibrium and diplotype analysis

Haploview 4.2 [35] was used to analyze the linkage disequilibrium among the SNPs. The diplotypes of the GWAS samples were estimated using fastPHASE 1.2 software [36] and BEAGLE 3.3.2 software [37,38].

### Imputation and evaluation of imputation accuracy

The genotypes of 33,303 SNPs were imputed using BEAGLE 3.3.2 software [37,38], with haplotype information inferred from 586,812 SNPs in 1041 Japanese Black cattle used as a reference.

The imputation accuracy was evaluated in 793 animals from the high-density dataset (BovineHD BeadChip) to assess the quality of the imputed genotypes. Genotypes were masked for all SNPs except the 33,303 SNPs corresponding to the Bovine SNP50K BeadChip (version 1). Genotypes for the 553,509 masked SNPs were inferred using BEAGLE 3.3.2, and imputed genotypes were compared with true genotypes using CalcMatch software developed by Yun Li [39].

### Expression analysis

For real-time quantitative PCR, we extracted total RNA from cow tissues using RNeasy mini kits (QIAGEN, cat#74104), and total RNA was treated with DNase I. The cDNA was synthesized from 50 ng RNA using ReverTra Ace-α (TOYOBO, cat#FSK-101) with random primers according to the manufacturer's instructions. *GTF2F2* was amplified with the following primers and probe: forward, 5’-gcggagaactcgacctgac-3’; reverse, 5’-agcccattgctgcgacaaa-3’; and probe, 5’-ttaggaaccttgaccagccacactccg-3’. Real-time PCR was performed on a 7900HT Real-Time PCR system (Applied Biosystems) using the comparative Ct method with *glyceraldehyde-3-phosphate dehydrogenase* (*GAPD*) as the internal control.

### Allelic imbalance test

To quantify the allelic imbalance of *GTF2F2* transcripts, we designed PCR primers to *BovineHD1200004611* (15465327 bp) on BTA12, located in the 3’ UTR of *GTF2F2* (Additional files 3 and 5). The forward primer was 5'-aaaacaggtggttgtgtctca-3', and the reverse primer was 5'-ccttacccctacaaccctcct-3'. We used 50 ng of template cDNA from primary dermal fibroblasts and ovaries and 10 ng of genomic DNA from heterozygous animals for PCR amplification with the TaKaRa Ex Taq HS DNA polymerase (TaKaRa, cat#RR006). The PCR product was directly sequenced. The peak height at the polymorphic site was quantified using PeakPicker 2 software [8]. Allelic imbalance was estimated as the ratio of the ratios of the peak height of the *Q* allele over the *q* allele in the cDNA and in the genomic DNA (gDNA) from the same animal. Calibration curves were generated using data obtained by mixing varying amounts of gDNA from *Q* and *q* homozygotes [9].

### Luciferase reporter assay

To measure the effects of the five SNPs and one indel within the upstream region (2,964 bp upstream of the start codon) and the nine SNPs within the 3' UTR of *GTF2F2* (935 bp) on transcription (Additional file 5), each haplotype (*Q* haplotype and *q* haplotype) was PCR amplified from gDNA. The following primers were used for the upstream region: forward primer (5'-GGGGTACCctatccatggggttctccag-3'; uppercase indicates the *Kpn*I linker) and reverse primer (5'-TCCCCCGGGgacctgcggacttagagcag-3'; uppercase indicates the *Sma*I linker). The following primers were used for the 3' UTR: forward primer (5'- GCTCTAGAgaagcccgcctaacagaact-3'; uppercase indicates the *Xba*I linker) and reverse primer (5'- GCTCTAGAaccatggacaggtattgttttt-3'; uppercase indicates the *Xba*I linker). The PCR products were digested with *Kpn*I-*Sma*I or *Xba*I and then cloned into the *Kpn*I-*Sma*I site of pGL3-basic (Promega, cat#E1751) or the *Xba*I site of pRL-TK (Promega, cat#E2241), respectively. The sequence of the insert and the direction were confirmed by sequencing. For cell culture, HeLa S3 cells were maintained in Dulbecco's modified Eagle's medium (DMEM) with 10% fetal calf serum (FCS) supplemented with 2 mM glutamine, penicillin (100 units/ml) and streptomycin (100 mg/ml). Bovine endometrial epithelial cells (BEnEpC, Cell Application, Inc., cat# B932-05) were maintained according to the manufacturer's instructions. Using Lipofectamine 2000 (Invitrogen, cat#11668-019), we transfected 1 × 10^5^ cells per well in a 24-well plate with a mixture of 400 ng of the reporter and 40 ng of pRL-TK Renilla or of pGL3-control firefly luciferase to calibrate transfection efficiency. The luciferase assay was performed 24 hr after transfection using the Dual Luciferase Reporter Assay system (Promega, cat#E1910) and GloMax (Promega).

### Replication study

For the replication study, we used 827 samples from the remainder of the cohort from the same farms used in the GWAS (Additional file 7). *BovineHD1200004575* (15,353,468 bp) and *BovineHD1200004611* (15,465,327 bp) were genotyped by direct sequencing of PCR products using each primer pair (Additional file 4). PCR products were sequenced using BigDye Terminator v.3.1 Cycle Sequencing Kits (Applied Biosystems), followed by electrophoresis using an ABI 3730 sequencer (Applied Biosystems), and typed using SeqScape V2.5 (Applied Biosystems).

### Estimation of the genetic variance explained by haplotype and effect size of haplotype

The effect of the haplotype were estimated as the least square mean values of generalized linear model (GLM) analyses. The statistical model for GLM analysis included fixed effects for the farm, birth year and haplotype. The genetic variance explained by the haplotype was calculated based on estimates of the haplotype effect and the frequency of the haplotype [40]. Total genetic variance was estimated by MTDF-REML. The effect size of a haplotype was estimated as the proportion of genetic variance explained by the haplotype.

## Abbreviations

GWAS: Genome-wide association study; NCP4: Number of calves produced at 4 years of age; QTL: Quantitative trait locus or loci; BTA: Bovine (*Bos taurus*) chromosome; SNP: Single nucleotide polymorphism; LD: Linkage disequilibrium; PCR: Polymerase chain reaction.

## Competing interests

The authors declare no conflicts of interest.

## Authors’ contributions

SS, TI, TW and YS designed the research; SS performed the GWAS analysis; TI collected and analyzed the data; SS and TI performed replication studies; SS, TM and SI performed expression analyses; and SS, TI and YS wrote the manuscript. All authors read and approved the final manuscript.

## Supplementary Material

Additional file 1 **Quantile-quantile plots of the genome-wide association results for NCP**_**4**_**.** The red dots represent the observed -log10 *P* values, and the straight line represents the expected -log10 *P* values under the null hypothesis. Click here for file

Additional file 2 **Accuracy of imputation.** Imputation accuracy was evaluated among 793 animals from the high-density dataset (BovineHD BeadChip) to assess the quality of the imputed genotypes. Genotypes were masked for all SNPs except for the 33,303 SNPs corresponding to the 50K BeadChip (version1, Illumina). Genotypes for the 553,509 masked SNPs were inferred using BEAGLE 3.3.2 [37,38], and imputed genotypes were compared with true genotypes using CalcMatch software [39]. The allelic discordance rate was calculated as follows: 1 - matched allele / 2 / masked genotype. The genotypic discordance rate was calculated as follows: 1 - matched genotype / masked genotype. Click here for file

Additional file 3 **The imputed SNPs associated with NCP**_**4 **_**on BTA12.** SNPs belonging to the BovineSNP50K BeadChip (version 1) are designated by *. The SNP positions are based on the UMD3.1 assembly of the bovine genome. The upper extremes and lower extremes correspond to NCP_4_ values above the 93.3^rd^ percentile and below the 6.68^th^ percentile, respectively. Click here for file

Additional file 4 **Primer information for *BovineHD1200004575* and *BovineHD1200004611.*** Click here for file

Additional file 5 **The positions of the variants in *GTF2F2 *and *KCTD4 *on BTA12.** The SNPs positions are based on the UMD3.1 assembly of the bovine genome. The upper extremes and lower extremes correspond to NCP_4_ values above the 93.3^rd^ percentile and below the 6.68^th^ percentile, respectively. Synonymous substitution is represented by *. *KCTD4* is located in intron 4 of *GTF2F2* and is transcribed in the opposite direction*.* Click here for file

Additional file 6 **Relative expression of *GTF2F2 *in cow tissues and cells.** Tissues and cells are indicated on the *y*-axis. Total RNA was extracted from tissues (1–17) and primary dermal fibroblasts (18) derived from two female Japanese Black cattle and from bovine primary endometrial epithelial cells (19). All samples and genes were analyzed in triplicate. Relative gene expression levels in the different tissues are shown as the mean quantity relative to the value obtained from the ovarian sample (dotted line). Click here for file

Additional file 7 **The distribution of NCP**_**4 **_**in 827 cows for the replication study.** A sample (n = 827) was derived from the remainder of the cohort from the same farms used in the GWAS. Click here for file

Additional file 8 **SNP information associated with NCP**_**4**_**.** SNP positions are based on the UMD3.1 assembly of the bovine genome. SourceSeq are SNP flanking sequences, which were provided by Illumina, Inc. Nucleotides in the SNPs are shown in brackets. Click here for file
