# Supplementary figures and images for: Variants in the 3' UTR of General Transcription Factor IIF, polypeptide 2 affect female calving efficiency in Japanese Black cattle
Source: BMC Genet. 2013 May 10;14:41. doi: 10.1186/1471-2156-14-41 (PMC3656791; doi:10.1186/1471-2156-14-41)

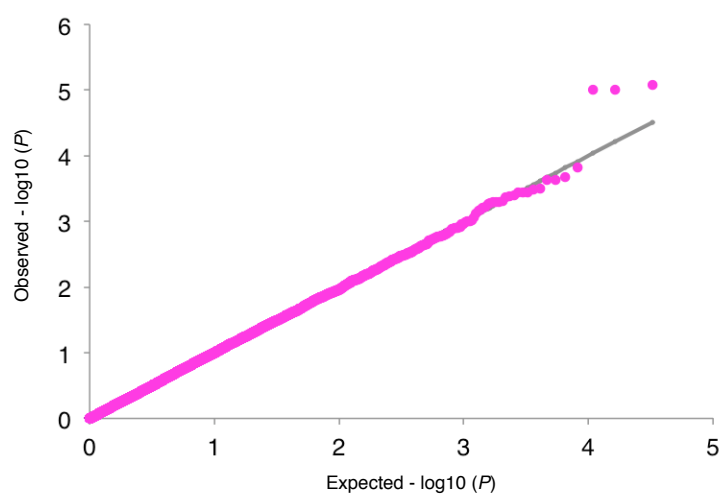

Supplement: Additional file 1 — Quantile-quantile plots of the genome-wide association results for NCP4. The red dots represent the observed -log10 P values, and the straight line represents the expected -log10 P values under the null hypothesis. [file 1471-2156-14-41-S1.pdf]

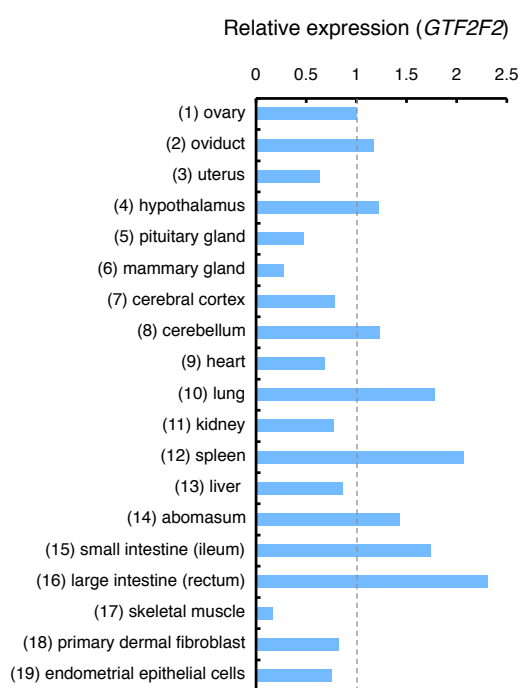

Supplement: Additional file 6 — Relative expression of GTF2F2 in cow tissues and cells. Tissues and cells are indicated on the y-axis. Total RNA was extracted from tissues (1–17) and primary dermal fibroblasts (18) derived from two female Japanese Black cattle and from bovine primary endometrial epithelial cells (19). All samples and genes were analyzed in triplicate. Relative gene expression levels in the different tissues are shown as the mean quantity relative to the value obtained from the ovarian sample (dotted line). [file 1471-2156-14-41-S6.pdf]

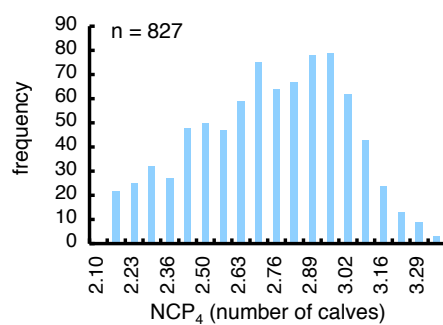

Supplement: Additional file 7 — The distribution of NCP4 in 827 cows for the replication study. A sample (n = 827) was derived from the remainder of the cohort from the same farms used in the GWAS. [file 1471-2156-14-41-S7.pdf]
